# Why West? Comparisons of clinical, genetic and molecular features of infants with and without spasms

**DOI:** 10.1371/journal.pone.0193599

**Published:** 2018-03-08

**Authors:** Anne T. Berg, Samya Chakravorty, Sookyong Koh, Zachary M. Grinspan, Renée A. Shellhaas, Russell P. Saneto, Elaine C. Wirrell, Jason Coryell, Catherine J. Chu, John R. Mytinger, William D. Gaillard, Ignacio Valencia, Kelly G. Knupp, Tobias Loddenkemper, Joseph E. Sullivan, Annapurna Poduri, John J. Millichap, Cynthia Keator, Courtney Wusthoff, Nicole Ryan, William B. Dobyns, Madhuri Hegde

**Affiliations:** 1 Epilepsy Center, Ann & Robert H. Lurie Children's Hospital of Chicago, Chicago, IL, United States of America; 2 Department of Pediatrics, Feinberg School of Medicine, Northwestern University, Chicago, IL, United States of America; 3 Department of Human Genetics, Emory University School of Medicine, Atlanta, GA, United States of America; 4 Department of Pediatrics, Children’s Healthcare of Atlanta, Emory University, Atlanta, GA, United States of America; 5 Department of Healthcare Policy & Research, Weill Cornell Medicine, New York, NY, United States of America; 6 Department Pediatrics, Weill Cornell Medicine, New York, NY, United States of America; 7 New York Presbyterian Hospital, New York, NY, United States of America; 8 Department of Pediatrics, University of Michigan, Ann Arbor, MI, United States of America; 9 Division of Pediatric Neurology, Seattle Children’s Hospital, Seattle, WA, United States of America; 10 Department of Neurology, University of Washington, Seattle, WA, United States of America; 11 Department of Neurology, Mayo Clinic, Rochester, MN, United States of America; 12 Departments of Pediatrics & Neurology, Oregon Health & Sciences University, Portland, OR, United States of America; 13 Department of Neurology, Massachusetts General Hospital, Boston, MA, United States of America; 14 Department of Pediatrics, the Ohio State University, Nationwide Children’s Hospital, Columbus, OH, United States of America; 15 Department of Neurology, Children's National Health System, George Washington University School of Medicine, Washington, D.C., United States of America; 16 Section of Neurology, St. Christopher's Hospital for Children, Drexel University College of Medicine, Philadelphia, PA, United States of America; 17 Department of Pediatrics and Neurology, School of Medicine, University of Colorado Anschutz Medical Campus, Aurora, CO, United States of America; 18 Division of Epilepsy and Clinical Neurophysiology, Boston Children’s Hospital, Harvard Medical School, Boston, MA, United States of America; 19 Department of Neurology, University of California San Francisco, San Francisco, CA, United States of America; 20 Cook Children’s Health Care System, Jane and John Justin Neurosciences Center, Fort Worth, TX, United States of America; 21 Division of Child Neurology, Stanford University, Palo Alto, CA, United States of America; 22 Departments of Neurology and Pediatrics, The Children's Hospital of Philadelphia, Philadelphia, PA, United States of America; 23 The Perelman School of Medicine at the University of Pennsylvania, Philadelphia, PA, United States of America; 24 Center for Integrative Brain Research, University of Washington, Seattle, WA, United States of America; 25 Seattle Children's Research Institute, University of Washington, Seattle, WA, United States of America; 26 Pediatrics University of Washington, Seattle, WA, United States of America; University of Modena and Reggio Emilia, ITALY

## Abstract

Infantile spasms are the defining seizures of West syndrome, a severe form of early life epilepsy with poorly-understood pathophysiology. We present a novel comparative analysis of infants with spasms versus other seizure-types and identify clinical, etiological, and molecular-genetic factors preferentially predisposing to spasms. We compared ages, clinical etiologies, and associated-genes between spasms and non-spasms groups in a multicenter cohort of 509 infants (<12months) with newly-diagnosed epilepsy. Gene ontology and pathway enrichment analysis of clinical laboratory-confirmed pathogenic variant-harboring genes was performed. Pathways, functions, and cellular compartments between spasms and non-spasms groups were compared. Spasms onset age was similar in infants initially presenting with spasms (6.1 months) versus developing spasms as a later seizure type (6.9 months) but lower in the non-spasms group (4.7 months, p<0.0001). This pattern held across most etiological categories. Gestational age negatively correlated with spasms onset-age (r = -0.29, p<0.0001) but not with non-spasm seizure age. Spasms were significantly preferentially associated with broad developmental and regulatory pathways, whereas motor functions and pathways including cellular response to stimuli, cell motility and ion transport were preferentially enriched in non-spasms. Neuronal cell-body organelles preferentially associated with spasms, while, axonal, dendritic, and synaptic regions preferentially associated with other seizures. Spasms are a clinically and biologically distinct infantile seizure type. Comparative clinical-epidemiological analyses identify the middle of the first year as the time of peak expression regardless of etiology. The inverse association with gestational age suggests the preterm brain must reach a certain post-conceptional, not just chronological, neurodevelopmental stage before spasms manifest. Clear differences exist between the biological pathways leading to spasms versus other seizure types and suggest that spasms result from dysregulation of multiple developmental pathways and involve different cellular components than other seizure types. This deeper level of understanding may guide investigations into pathways most critical to target in future precision medicine efforts.

## Introduction

Infantile spasms (IS) are the defining seizures of West syndrome, an early life epilepsy (ELE) associated with refractory seizures, severe developmental consequences and early mortality. West syndrome is characterized by a specific electrographic signature, hypsarrhythmia, a highly chaotic pattern characterized by multifocal spikes and high voltage slow waves. Additionally, West syndrome involves developmental delay although this feature is not necessarily present from the outset. Its core feature, however, is the seizure type, the epileptic (or in infants, infantile) spasm itself, which does not resemble other cortically-generated seizures and has a distinct ictal pattern on the electroencephalogram (EEG), a high voltage slow wave followed by an electro-decrement. Subcortical mechanisms are likely involved in its generation [1–3]. For clinical purposes, the seizure type, not the syndrome, is the focus of evidence-based treatment guidelines. Notably, the current best treatments involve use of steroids or the hormone, ACTH (not a standard anti-seizure medication) or vigabatrin (not considered a first line anti-seizure medication except for treatment of IS) [4–6].

Causes of IS, and of epilepsy in general, are numerous and include acquired as well as congenital factors such as brain malformations, in-born errors of metabolism, tuberous sclerosis complex (TSC), and multiple different genetic factors ranging from point mutations in individual genes to large copy number variants involving multiple genes and whole chromosomes [1, 3, 7–14]. Each individual factor, especially individual genes, is rare rendering comparative analyses of potentially hundreds of genes cumbersome and often uninformative. The current literature in this area rests on reports of one or a few patients with variants in specific genes. Occasionally, a larger multi-center series is assembled over many years. This approach has been invaluable, and research under this paradigm will likely continue; however, to move from gene discovery to underlying biology is a needed next step. Bioinformatics offers tools, such as gene ontology, gene set enrichment and molecular pathway analysis (GO-pathway analysis), to query large gene datasets and reduce the genetic heterogeneity by identifying common molecular pathways and functions that might unite multiple rare genetic variants.

To our knowledge, no prior study, has explicitly investigated whether or how etiologies identified in infants with spasms are distinct from those of other epilepsies occurring at the same age. Consequently, the specificity of associations between IS and various etiologies is unclear. Although gene set enrichment and pathway analysis has been used in the autism literature, we are unaware of its application to compare two different clinically defined groups in an effort to identify underlying biological differences between them.

A direct comparison of the clinical and molecular factors associated with IS versus with other ELE can help identify underlying mechanistic differences distinguishing them. Such knowledge could facilitate identification and development of new therapeutic approaches for IS or even IS prevention in at risk infants. Disease model development could also be guided by such information.

To provide insights into the causes specifically related to IS, we compared clinical, genetic, and molecular factors in children who developed IS to those who developed other ELE within a clinical-epidemiological framework.

## Materials and methods

### Study subjects and inclusion criteria

**Fig 1:**
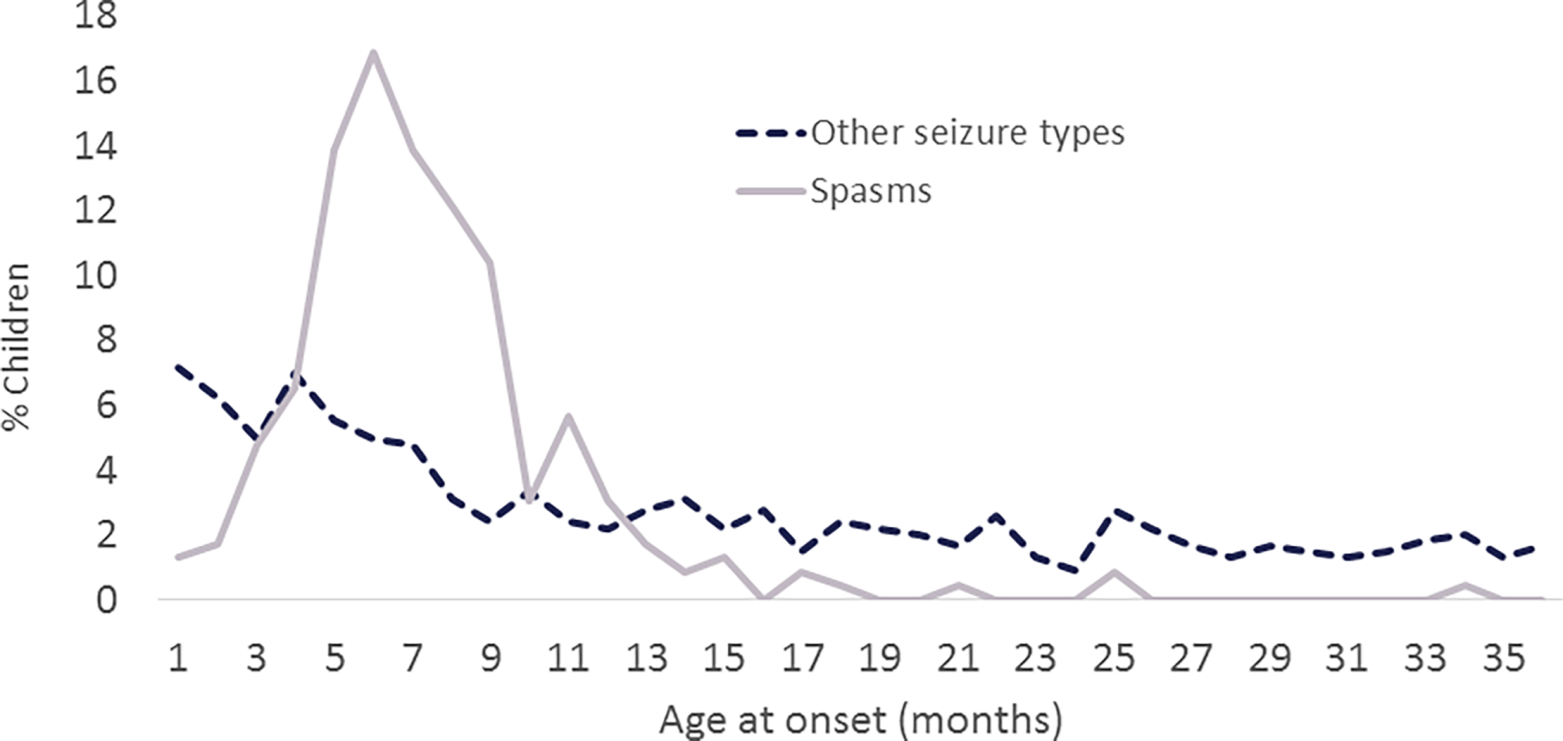
Age at seizure onset. Age at onset of epilepsy by initial seizure type, infantile spasms versus other ELE.

The Institutional Review Board of the Ann & Robert H Lurie Children's Hospital of Chicago reviewed and approved the protocol. The IRBS of each institution that recruited patients also reviewed and approved the protocols for recruitment of children's at each site. These are: the Oregon Health Services University, Seattle Children’s Hospital, New York Presbyterian Hospital, University of California, San Francisco Beniof Children’s Hospital, Mayo Clinic, C.S. Mott’s Children’s Hospital, Nationwide Children’s Hospital, National Children’s Medical Center, Johns Hopkins All Children’s Hospital, St. Christopher’s Hospital for Children, Colorado Children’s Hospital, Lucile Packard Children’s Hospital, Cook Children’s Health Care System, Children’s Hospital of Philadelphia, Boston Children’s Hospital, and Massachusetts General Hospital. Data are from a prospective, observational cohort study conducted primarily by chart review. Children with newly-presenting epilepsy, initially evaluated and diagnosed from March 2012 through April 2015 at 17 US-based pediatric epilepsy programs participating in the Pediatric Epilepsy Research Consortium (PERC) were the target population. The study targeted children who had their first seizure before their third birthday and who had not established epilepsy care elsewhere prior to their evaluation at the participating center. We included children who were initially diagnosed and treated in the emergency department or by a primary care physician, so long as referral was made at that time to the participating hospital for full evaluation. The initial stages of analysis demonstrated that almost all (93%) of children who developed IS (either as the presenting seizure type or during the year after initial diagnosis) had their epilepsy onset in the first year of life. By contrast, only 54% of all other children in our cohort without IS had onset of epilepsy during the first year of life (Fig 1). Consequently, analyses are limited to those children with onset of seizures before their first birthday. Informed, written consent was obtained from the parents of the children.

### Clinical data

Medical records from the initial diagnostic evaluation and through one year after were reviewed. We included neonates with unprovoked seizures (neonatal epilepsy); children who met very early criteria for specific forms of epilepsy (e.g. Dravet syndrome) and those with a single seizure if, in the judgment of the treating physician, they were at high risk of further seizures and medication was initiated [15]. Age at first spasm was separately recorded if it was not the first seizure type. Seizure types and electroclinical syndromes were determined by pediatric epileptologists at each site based on standard criteria [16]. Etiological, seizure, and epilepsy syndrome diagnoses were based on all information collected through one year following initial diagnosis. Approximately 100 IS patients in our cohort were previously included in a preliminary report that focused only on children with IS and was limited to information collected at initial assessment and up through three months after [14].

This was a strictly observational study; all evaluations and treatment decisions were performed per the treating physicians. Etiology was determined from history, physical exam, neuroimaging, metabolic and genetic testing performed before, at, and through one year after diagnosis. Etiology was grouped as follows: focal cortical dysplasia (FCD), other brain malformations, tuberous sclerosis complex (TSC), other neurocutaneous diseases, hypoxic-ischemic encephalopathy (HIE), intraventricular hemorrhage (IVH), other acquired injuries, metabolic diseases, dysmorphic syndromes that are clinically recognizable without genetic testing (e.g. Down syndrome) but for which confirmatory genetic testing is typically performed, genetic causes identifiable only from genetic testing (e.g. a pathogenic *SCN1A* variant), other specific causes, and cause unknown. Genetic testing was often performed to clarify the molecular basis of clinical diagnoses such as TSC, other neurocutaneous diseases, brain malformations, and neurometabolic diseases.[17]

### Genetic and molecular pathway analyses

We used the results of the genetic testing ordered by the treating physicians. Tests included classical karyotypes, chromosomal microarrays (CMA), single gene testing, targeted gene panels, mitochondrial function genes, and whole exome sequencing (WES). All testing was performed in CLIA (Clinical Laboratory Improvement Amendment) and CAP (College of American Pathologists) certified facilities and interpretations made by board-certified geneticists according to the American College of Medical Genetics and Genomics (ACMG) and Association for Molecular Pathology (AMP) guidelines. Although the genetic tests performed within this cohort were for clinical purposes only and consequently varied somewhat across centers, the interpretation of pathogenicity was performed in accordance with ACMG-AMP guidelines by board-certified molecular geneticists at the labs that performed the analyses. A full account of pathogenic variants found in this cohort is available [17].

We used the MSigDB software and databases from the Broad Institute to perform gene ontology and pathway analysis (GO-pathway analysis). This technique identifies enriched gene sets for one or more shared common biological features (pathways, functions and cell compartments) that are statistically significantly enriched in the gene list of interest (p<0.01; false discovery rate (FDR)<0.05) based on greater than 1325 biologically defined gene sets [18–20]. Only genes harboring variants classified as pathogenic or likely pathogenic following ACMG-AMP guidelines [21] in CLIA-CAP-certified laboratories were included. We did not include genes harboring variants of uncertain significance (VUS). In the case of copy number variants (CNVs), we included in the analyses those genes in the region affected by the CNV if the gene appeared in the Courtagen’s epiSEEK comprehensive 471 epilepsy and seizure disorders gene panel (S1 Table**)**. CNVs in which a candidate gene(s) could not be identified were excluded from GO-pathway analysis. Due to their strong association with Down syndrome, *DYRK1A*, *RCAN1*, *PSMG1*, *DSCR3*, *DSCR4* genes were included for patients with trisomy 21 [22, 23]. We used a total of 50 genes observed in 92 children as input in our pathway analysis (S2 Table). Enrichment analysis was performed using the following gene sets from different databases in the MSigDB software: for biological pathways, we included hallmark gene sets (H), curated gene sets (C2) [that further includes chemical and genetic perturbations gene-sets (C2:CGP), canonical pathways (C2:CP), gene-sets from BioCarta pathway database (C2:CP:BIOCARTA), KEGG pathway database gene-sets (C2:CP:KEGG), REACTOME database gene-sets (C2:CP:REACTOME)], microRNA target motif gene sets (C3:MIR), cancer modules gene sets (C4:CM), gene ontology biological processes gene sets (C5:BP), oncogenic signatures gene sets (C6), and immunological signatures gene sets (C7); for cellular compartments and molecular function analysis, we included gene ontology cellular compartments gene sets (C5:CC), and gene ontology molecular functions gene sets (C5:MF), respectively.

We performed GO-pathway analysis for biological processes (pathways), cellular compartments and molecular functions to identify the top 50 statistically significant (p<0.05, FDR q<0.05) enriched gene sets. This pathway analysis typically results in numerous enriched “raw” gene sets for analysis. To reduce redundancy across these enriched genes sets, we consolidated them by manually combining the initial gene set categories into compiled categories based on a) established hierarchical superfamily of the gene ontology (GO) functions [24], and b) biological similarity of the individual enriched gene-sets based on the name, definition and type of the individual gene sets (S3 Table). Finally, we performed analyses in which we directly compared children with and without IS with respect to the compiled pathways, cellular compartments, and molecular functions identified in the GO-pathway analysis.

### Statistical analyses

Statistical analyses were conducted in SAS (Cary, NC). To compare means for gestational age, we used student’s t-tests. Chi-square tests were used to test associations between dichotomous variables. We used Pearson correlations to test the associations between gestational age and seizure onset age separately within groups defined by the presence and absence of spasms. Analysis of covariance (ANCOVA) was used to adjust age at onset of seizures for gestational age. Within the ANCOVA model, the F-test was used to test differences in onset age for children with vs without spasms within each etiology group. To compare the prevalence of each of the compiled enriched gene set categories in children with versus without spasms, chi-square tests were used with FDR correction. For graphic presentation, the prevalence ratio with 95% confidence interval was calculated. Only compiled categories affecting genes in 10 or more children were included to assure a meaningful number of patients available for analysis. A correction for FDR was employed to adjust for multiple comparisons with q<0.05 as the critical value [25].

## Results

The original cohort enrolled 775 children of whom 509 (65.7%) had the initial onset of epilepsy in the first year of life. The 509 infants included 261 (51.3%) boys, and the average age at first seizure was 5.2 months (standard deviation (SD) = 3.0; median = 5.1m interquartile range (IQR) = 3.0–7.2).

Infantile spasms occurred as the presenting seizure type in 212 (42%) infants, and 41 (8%) who presented with other seizure types evolved to have IS over the course of follow-up. The remaining 256 (50%) infants did not have any IS during the period of observation. Forty-eight infants, who did not develop IS, were not followed to their first birthday, eight of whom died. Another two with onset in the first year of life evolved to IS at 13 months. These 50 infants were retained in the analysis as not having IS (N = 48) and as developing IS (N = 2) respectively.

### Age of onset

**Fig 2:**
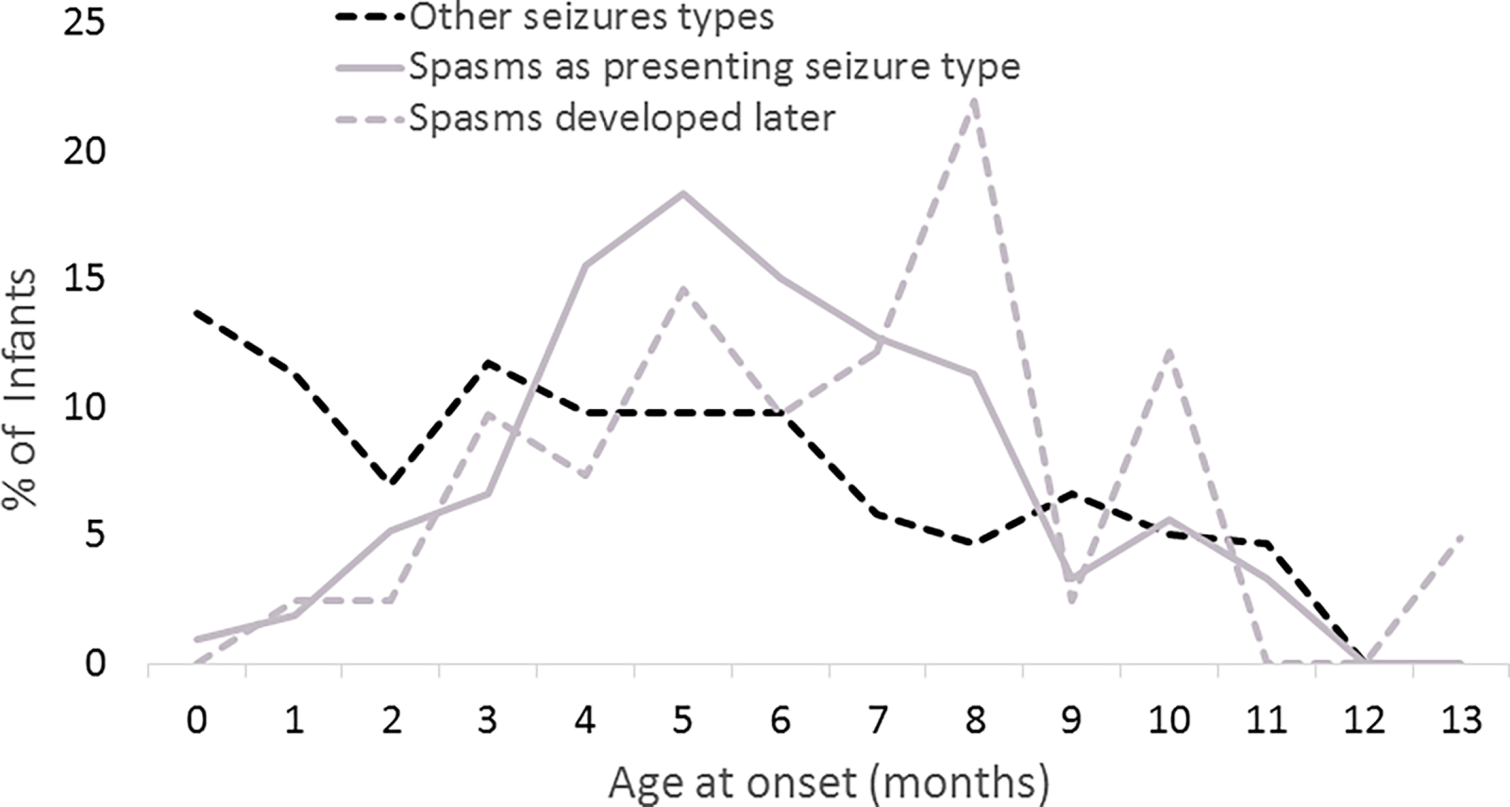
Age at onset of infantile spasms as the initial seizure type and as a later seizure type and age at onset of initial seizure who never developed spasms for infants with initial onset of epilepsy in the first year of life.

The age of IS onset was similar in infants who initially presented with IS (spasms age = 6.1m, CI = [5.8, 6.4]) and in those who initially presented with other seizures and later evolved to IS (spasms age = 6.9m, CI = [6.0–7.8], p = 0.05, Fig 2). By contrast, the age of onset of other seizures for children who never had IS during the observation period was younger (4.7m, CI = [4.3, 5.1]) compared to children with IS at onset (p<0.0001, t-test) and children who developed IS after first presenting with other seizure types (p = 0.03). The age at initial seizure onset in infants who later evolved to have spasms was 3.5m (SD = 2.3, IQR = 2.0, 4.7)).

Gestational age had a strong inverse association with age at IS onset (Pearson correlation = -0.29, p<0.0001) but not with onset of non-spasms seizures (Pearson correlation = 0.04, p = 0.54). The direction of the effect indicated that the more premature an infant at birth, the greater was the chronological age at spasms onset. This correlation was found to be absent in the other seizure types.

### Clinically determined etiology

Evaluations used to determine etiology included neuroimaging (MRI, N = 479, 94%; CT N = 5, 1%), and various metabolic (N = 326, 64%) and genetic (N = 266, 52%) tests. Intensity of investigations was somewhat greater in children with than without IS (S4 Table). With the unknown etiology group as the reference, acquired injuries (hypoxic-ischemic encephalopathy (HIE), intraventricular hemorrhage (IVH), and others), TSC, and dysmorphic syndromes (largely Down syndrome) were more commonly associated with IS than with other forms of epilepsy (Table 1). After FDR correction, these associations remained statistically significant with the exception of HIE. The presence of FCD, other brain malformations, metabolic disorders, other neurocutaneous syndromes, and other genetic causes (as a group) was comparable in children with and without IS; none was statistically significant.

**Table 1:** Etiology and association with occurrence of spasms in infants. Chi-square test was used to compare each category to unknown category as the reference.

Etiology	Total	No spasms (N = 256)	Spasms (N = 253)	p-value*
**Focal cortical dysplasia**	11 (2%)	5 (45%)	6 (55%)	0.34
**Other brain malformations**	73 (14%)	38 (52%)	35 (48%)	0.24
**Tuberous sclerosis**	17 (3%)	3 (18%)	14 (82%)	0.0007***
**Other Neurocutaneous**	10 (2%)	6 (60%)	4 (40%)	0.99
**Hypoxic-Ischemic Encephalopathy**	19 (4%)	7 (37%)	12 (63%)	0.05
**Intraventricular hemorrhage**	26 (5%)	8 (31%)	18 (69%)	0.005***
**Other acquired**	34 (7%)	11 (32%)	23 (68%)	0.003***
**Metabolic**	15 (3%)	6 (40%)	9 (60%)	0.13
Clinical dysmorphic syndromes**	36 (7%)	10 (28%)	26 (72%)	0.0003***
**Other Genetic**	41 (16%)	27 (66%)	14 (34%)	0.47
**Other**	15 (3%)	8 (53%)	7 (47%)	0.62
**Unknown**	212 (42%)	127 (60%)	85 (40%)	Reference group

* Each etiology group was compared to the unknown etiology group-value based on Chi-square test.

** Down Syndrome (N = 22), Wolf-Hirschorn (N = 4), 1 each of 10 other syndromes.

***Chi-square test statistically significant after Benjamini-Hochberg correction for false discovery rate (p = 0.02)

### Age of onset and etiology

Because some acquired injuries (notably IVH and HIE) occur more often in premature infants, we adjusted for gestational age using linear regression and compared the adjusted onset age of IS (as initial or later seizure type) to that of onset age in children who never developed spasms. We excluded the age at onset of non-spasm seizures in the 41 children who later developed IS and only counted them once in the IS group. For all but one etiology (FCD), age was older in the IS than in the non-IS group, significantly so overall and for other brain malformations, IVH, and other acquired injuries (q<0.05, Table 2). Notably, the age at spasms onset was reasonably consistent across the etiology groups with the exception of FCDs. The same was true for age at onset of seizures in the non-spasms group.

**Table 2:** Gestational-age adjusted age at onset by etiology and seizure type in infants (first seizure before the first birthday). Adjusted gestational ages in children with and without spasms testing with F-test in an ANCOVA model with in each etiology group*.

Etiology	ANCOVA-adjusted gestational age and standardized to 40 week gestational age		p-value**
	No spasms during observation period (N = 255) Mean (95% CI)	Spasms initially or later (N = 251) Mean (95% CI)	
**Total**	4.8 (4.4, 5.1)	6.2 (5.8, 6.5)	<0.0001***
**Focal cortical dysplasia (N = 11)**	3.7 (0.8, 6.6)	3.5 (1.3, 5.7)	0.88
**Other brain malformations (N = 73)**	3.6 (2.6, 4.7)	6.0 (5.1, 6.9)	0.001***
**Tuberous sclerosis (N = 17)**	3.5 (0, 7.2)	6.7 (5.4, 7.9)	0.10
**Other Neurocutaneous (N = 10)**	4.6 (2.8, 7.1)	4.9 (2.6, 7.1)	0.79
**Hypoxic-Ischemic Encephalopathy (N = 19)**	5.0 (2.6 (7.5)	6.8 (5.4, 8.1)	0.25
**Intraventricular hemorrhage (N = 26)**	4.1 (1.6, 6.5)	6.2 (5.0, 7.5)	0.01***
**Other acquired (N = 34)**	3.2 (1.3, 5.2)	6.6 (5.5, 7.6)	0.0003***
**Metabolic (N = 15)**	2.4 (0, 5.1)	5.3 (3.6, 7.0)	0.05
**Clinical dysmorphic syndromes (N = 34)**	6.0 (3.8, 8.2)	6.2 (5.3, 7.1)	0.97
**Other Genetic (N = 41)**	4.9 (3.7, 6.2)	6.1 (4.7, 7.5)	0.37
**Other (N = 15)**	5.4 (3.1, 7.7)	5.6 (3.3, 7.8)	0.26
**Unknown (N = 211)**	5.2 (4.6, 5.8)	6.0 (5.5, 6.5)	0.13

*3 children had missing GA and are excluded from GA-adjusted analyses

**For each etiology group, comparison of ages of onset for children who never developed spasms during the observation period and those who did. F-test based on a multiple linear regression analysis.

***Even with adjustment for multiple comparisons using Benjamini-Hochberg False Discovery Rate (FDR) q<0.05 procedure, these p values remain statistically significant.

### Molecular-genetic associations with infantile spasms

For purposes of genetic analyses, we excluded 79 infants whose conditions were attributed to acquired insults. One or more forms of genetic testing was performed in 251 (58.3%) of the remaining 430 of the infants. Tests in these 251 infants included classical karyotyping (N = 48), CMA (N = 145), epilepsy gene panels (N = 90), WES (N = 28), mitochondrial function gene analyses (N = 15), and a variety of other targeted single or multiple gene tests (N = 69). Some children had multiple types of testing performed. Pathogenic variants were found in 117 (46.6%) of those tested. Of these, 109 were interpreted as definitively diagnostic, and eight cases had heterozygous pathogenic variants in genes typically associated with autosomal recessive inheritance. In six of these eight cases, the phenotype definitely or potentially matched the disease associated with the gene, several of which have been reported occasionally to segregate in an autosomal dominant manner. These included variants in *NDUFAF5*, *RELN*, *FUCA1*, *SAMHD1*, *POLG*, *SCO2*. We included these six genes in our GO-pathways analysis. Sixteen children had large copy number variants for which specific candidate genes could not be identified. Another 17 children carried clinical diagnoses of specific genetic conditions (Down syndrome (N = 6), TSC (N = 9) and one each of neurofibromatosis and “Kabuki” syndrome) but without clinical laboratory genetic confirmation. These 33 children were also excluded from the GO-pathway analysis leaving 92 children in the analyses.

Most genes harboring pathogenic variants in this cohort were seen in only one or two children (S2 Table). The genes for which pathogenic variants were identified in the most number of infants, *SCN1A* (N = 10) and *PRRT2* (N = 4), were identified only in children without IS. IS was present in most of the children with pathogenic variants in *CDKL5* (3/4) and *TSC2* (5/7) and with Down syndrome (with or without karyotype confirmation of trisomy 21) (20/22).

### Pathway and cellular compartment enrichment analysis show a “bigger” impact of broad neurodevelopmental and regulatory pathways in infantile spasm than other seizures

**Fig 3:**
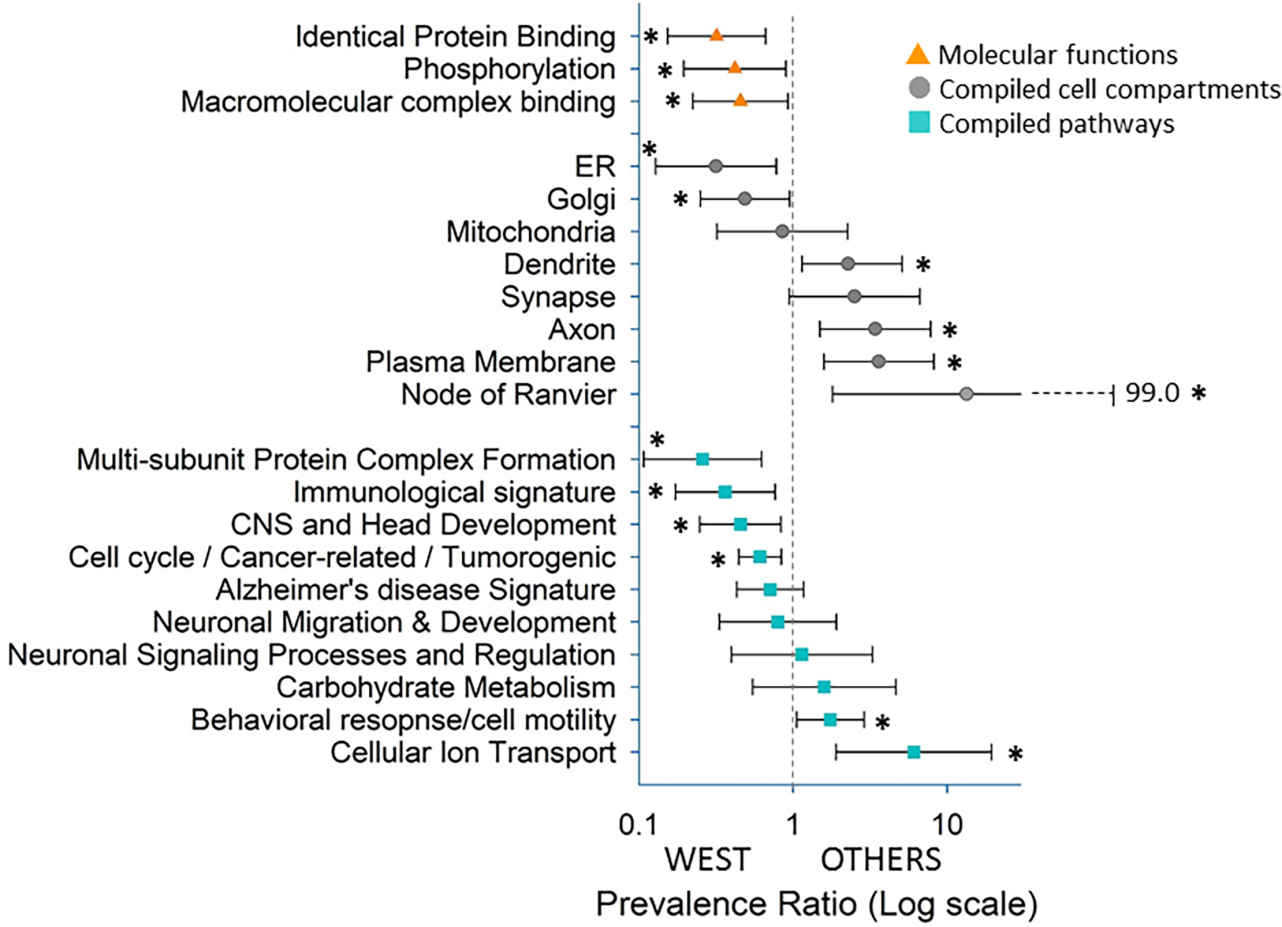
Gene Ontology (GO)-pathway analysis. Prevalence ratio showing significant association by Chi-square test of enriched compiled biological processes (pathways) (blue squares), cellular compartments (grey circles), and molecular functions (yellow triangles) to either infantile spasms group (West) or other epilepsies non-spasms group (Others). X-axis is displayed on a log scale. Significant association (q<0.05) is denoted by asterisk (*).

**Fig 4:**
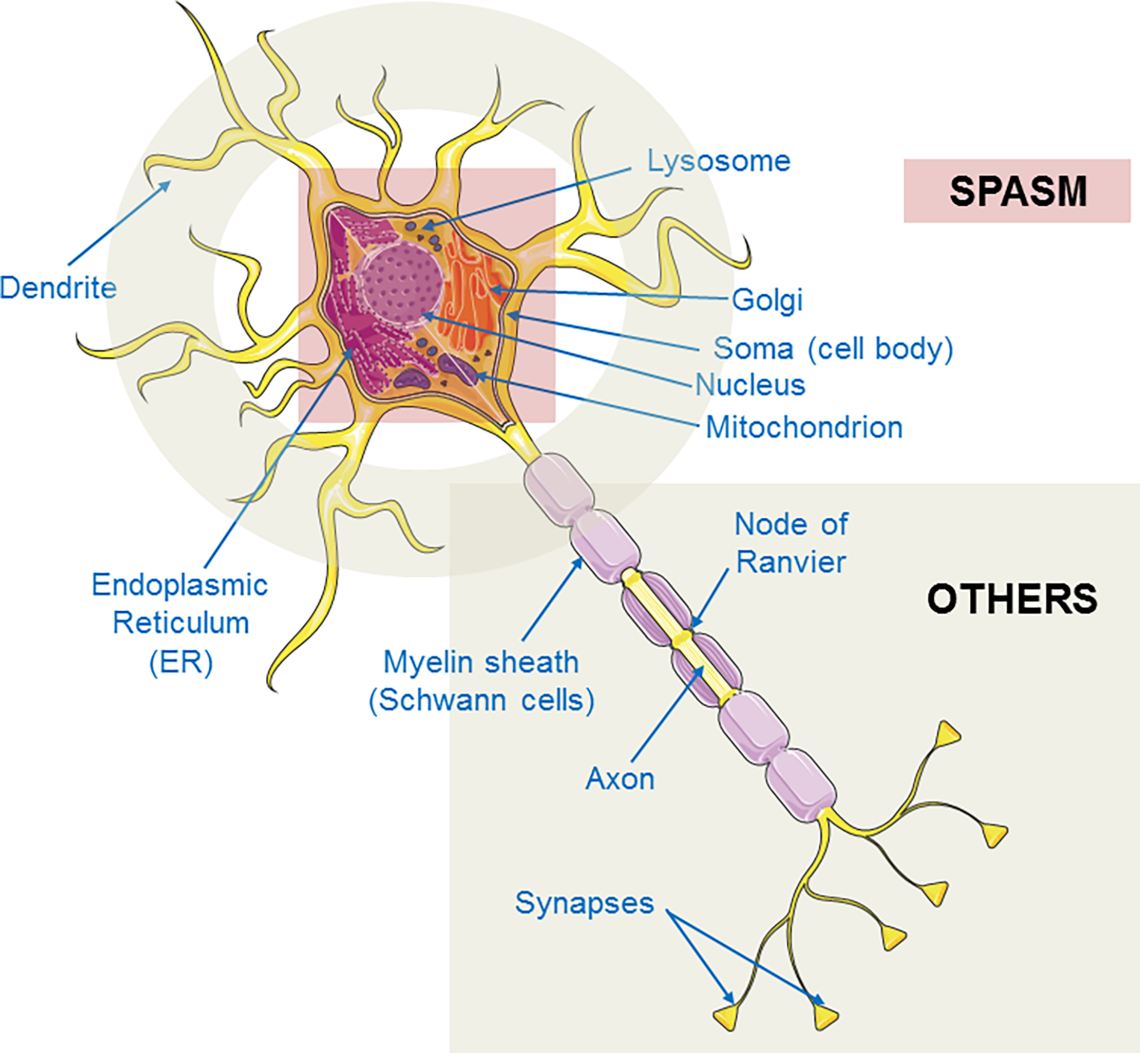
Cellular compartment association to spasm and other seizures. A schematic diagram of a neuron showing association of enriched compiled cellular compartments to either infantile spasms group or other epilepsy group. Golgi and endoplasmic reticulum (ER) and overall cell body (soma) are significantly associated with the spasms group. Dendrites and axonal regions including Node of Ranvier are significantly associated with other epilepsies.

We performed GO-pathway analyses on the genes with pathogenic variants. The analyses identified the top 50 statistically significant (FDR q<0.05) biological processes (pathways), 38 cellular compartments, and three molecular function groups for genes with pathogenic variants in our cohort. Because of the large number of enriched pathways and cellular compartments, many of which were overlapping and redundant, we compiled the functional pathway and cellular compartments into broader categories based on their hierarchical and biological relationships (S3 Table). As there were only three molecular function groups, we did not compile these further (although they are still referred to as compiled functions below for simplicity). Comparisons were then made between children who presented with or developed IS during the observation period (spasms group) to those who did not have IS during that period (non-spasms group) with respect to each of these compiled categories (Fig 3). Comparisons were limited to compiled categories that affected at least 10 infants. Of the eight compiled enriched cell compartment categories, six were significantly associated with seizure type (spasms vs. non-spasms) (q <0.05). Of the 10 compiled enriched biological pathways six were significantly associated with seizure type after FDR correction. These comparisons indicated a substantially higher representation of broad developmental pathways focused on central nervous system, cell cycle regulation and tumorigenic pathways (including microRNA regulation by miR150 and miR200A), and broad immunological processes in the spasms compared to non-spasms group. By contrast, in the non-spasm group, cellular behavioral responses due to stimuli and motor activity pathways such as cellular motility appear preferentially implicated. These include cellular ion transport, cell motility, and motor functions. In comparing cellular compartments between the spasms and non-spasms groups, we found that basic cellular organelles of the neuronal cell body, such as Golgi and endoplasmic reticulum (ER), were preferentially implicated in the spasms group. By contrast, axonal and synaptic regions, node of Ranvier, dendrites and plasma membrane are preferentially implicated in the non-spasms group (Fig 4). The three enriched molecular function categories reflected protein-protein interactions, molecular complex formations, and enzyme activity, in particular kinase activity for cellular protein phosphorylation. All three were preferentially and significantly associated with IS.

We directly compared the biological pathways for more commonly affected genes. None of the enriched sets of pathways and functions associated with *SCN1A* was associated with the Down syndrome genes (*DYRK1A*, *PSMG1*, *RCAN1*, *DSCR3*, *DSCR4*) or with TSC2. Notably, there was little overlap in the compiled biological pathways between TSC2 and the Down syndrome genes.

## Discussion

We provide a novel head-to-head comparison of infants with and without IS to identify clinical factors and molecular differences that may help elucidate the unique pathophysiology of spasms.

Analyses demonstrates the unique age structure of IS compared to other epilepsies. The onset age of IS has been previously recognized [1, 26, 27] but without direct comparison to other ELE. Our age finding was not due to a single etiological factor that was particularly common in the IS group, and onset age of IS was reasonably consistent across most etiological groups. Spasms onset age tended to be older than non-spasms onset age within a given etiological group. Further, the age at spasms onset was very similar in children initially presenting with spasms (6.1 months) and in those developing spasms as a later seizure type (6.9 months). By contrast, the onset age of other seizures is more broadly distributed. Finally, the finding of a moderate correlation between gestational age and spasm onset suggested the need to reach a certain post-conceptional stage in neurodevelopment (to catch-up), not just chronological age, before the developing brain can manifest IS. By contrast, gestational age was not associated with onset age for other seizure types beginning in the first year of life.

IS (West syndrome) have been the subject of numerous studies and occur in association with multiple etiological factors [7, 8, 11–14, 28]. Recent studies have sought to identify genetic variants associated with IS. For example, one study utilized comparative genomic hybridization arrays, whole exome and targeted sequencing, and described a broad genetic spectrum associated with IS; however, there was no comparison with other ELE [29]. The epi-4K study utilized exome sequencing to create protein-protein networks in IS but without any comparison with other ELE [12]. Paciorkowski et al. performed gene ontology-based gene set or pathway enrichment analysis of genes associated with IS and suggested that pathogenesis involved errors in molecular networks of GABA-ergic forebrain development. While intriguing, there was no comparison group without IS [30]. Despite these efforts, the unique pathophysiology of IS remains elusive.

Our approach differs substantially from the gene discovery literature because it was performed within a defined clinical epidemiological context and included all children from the point of initial diagnosis of epilepsy, regardless of cause. This is important as most recent research has focused only on novel gene discovery without accounting for two of the most common causes of IS, trisomy 21 and TSC. Further, many malformations of the brain—most are of genetic origin—are also associated with spasms but are typically excluded from the gene-finding literature. Inclusion of the fully array of factors associated with early life epilepsy in our analyses facilitated several comparisons of key clinical, genetic, and molecular features.

By using data reduction techniques (GO-pathway analysis) based on established methods, we extracted underlying biological commonalities shared by the 50 different genes affected in our sample. These extracted “variables” were then used in analyses to compare children with and without IS. The most common genetic finding was trisomy 21 (N = 17); the pathways and cell compartments associated with this, however, were also associated with a much larger number of non-chromosome 21 genes. The molecular features shared by trisomy 21 genes and other genes then became the subject of analysis rather than the genes themselves. This greatly enhanced statistical power for comparative analyses between infants with and without IS.

Our analyses indicate several intriguing differences between IS and other ELE. Pathway enrichment analyses suggest that molecular factors implicated in broad neurodevelopmental and regulatory mechanisms were more preferentially associated with IS. This finding provides a potential mechanistic explanation for the heuristic developmental desynchronization hypothesis [1] and is reflected in the distinct clinical pattern of onset age, which, unlike other seizures occurring in infancy, is centered around 6 months (Figs 1 and 2). Of note, the developmentally comparable age in the primate rhesus monkey (6 days is equivalent to ~6 months in the human infant based on brain growth spurt timing [31], is when expression changes occur in the greatest number of genes in the brain [32]. Thus the time of spasms onset coincides with the time when a virtual storm of transcriptional changes is occurring in the developing brain.

Analysis of cellular compartment gene sets demonstrates that genes whose functions are most expressed in the cell body, Golgi and ER were preferentially associated with IS. By contrast, genes primarily expressed in the axon, synapse, and dendrite were preferentially associated with other seizure types (Fig 4). This was not due solely to associations with *SCN1A*, a voltage-gated sodium channel gene expressed in the axon initial segment, but reflected common functions shared among several genes.

Regulatory targets of miRNAs with functions in cell growth, proliferation, tumors and oxidative stress response (miR200A [33–36]) and immune development (miR150 [37]) were preferentially enriched in infants with IS (S3 Table). miRNAs are active throughout life. They play an important regulatory role in neurodevelopment and neuropathology [38–40] and may also play a role in epileptic seizures [41, 42]. The preferential association of the miRNA enrichment seen in infants with spasms compared to those with other seizure types may reflect a role of epigenetic regulatory mechanisms in the pathophysiology of spasm. Microglia, immune molecules such as cytokines and chemokines are also known to be involved in synapse pruning during brain development.[43–46]

Our clinical comparisons demonstrated trisomy 21, TSC, and early acquired injuries were preferentially associated with IS. Acquired injuries generally do not originate directly from a neurogenetic factor. Recent evidence, however, shows that activity of 80% or more of cellular functions and pathways are altered in response to brain injury [47]. Given the significant representation of cellular and molecular pathways related to neurodevelopmental, cell cycle and growth pathways in IS, one could speculate that pathways involved in response to acquired injuries that lead to IS share similar mechanism affected by pathogenic variants in IS-associated genes.

Some limitations of this study include that genetic testing was not done under a uniform protocol and ultimately reflected the clinician’s preference. The results of those assessments, however, are based on the highest standards of variant interpretation by board-certified geneticists following evidence-based classification and ACMG-AMP guidelines. Consequently, we can be confident that the variants that we did include are likely explanations for the patients’ diseases.

The cohort was not population-based but hospital-based. Consequently there is some potential for over representation of infantile spasms; 50% with spasms is somewhat more than we might expect based on more population-based studies [48]. Follow-up was only for one year after initial diagnosis of epilepsy, and some children died or were lost. Consequently, we may have missed a few children who developed spasms. While later-onset spasms are of interest, it is unclear if they represent the tail end of the spectrum of IS or a different phenomenon. To minimize potential heterogeneity, we focused on the first year of life.

Strengths of our study include that the cohort targeted newly diagnosed children and was not limited based on type of underlying cause. Consequently, our data come closer to approximating the distribution of etiologies in new-onset ELE in the population than do gene-discovery studies. Notably, we included well-known and common causes of IS (trisomy 21, *TSC2*) and children with brain malformations and neurometabolic diseases which are typically excluded from gene discovery investigations.

All genetic variants were reported through CLIA-CAP-certified labs and their interpretations conformed to ACMG-AMP standards [21]. By contrast, the Epi4K study, one of the largest epilepsy gene discovery ventures yet, while using stringent statistical criteria to implicate genes associated with IS and Lennox-Gastaut Syndrome, still relied on *in silico* prediction algorithms for interpretation of pathogenicity [12]. Requiring CLIA-CAP-certification of pathogenicity may be overly conservative; however, it reduces noise by focusing only on genes with pathogenic variants whose contribution to phenotype reaches a high level of certainty and excludes variants of uncertain significance with inadequate evidence of functional impact.

Our findings have three main implications: (1) Many infants at risk of developing IS are identifiable before spasms occur (trisomy 21, TSC, acquired injuries, onset in the first months of life). Efforts to prevent spasms in babies with TSC are already underway.[49–52]. To our knowledge, no such efforts exist for trisomy 21. (2) Much effort goes into developing animal models of IS in which to test new therapies. Our findings suggest molecular-neurodevelopmental aspects that need to be present in such models to emulate the human condition. (3) Our findings represent an initial application of bioinformatics analysis in a clinical-epidemiological context to answer the clinical question: why do some children develop West syndrome/infantile spasms? ELE represent individually rare disorders with tremendous genetic heterogeneity. Headway in developing effective therapeutics is slow, in large part because of how rare these disorders are. Next generation sequencing (NGS)-based technologies coupled with tools such as GO-pathway analysis, provides a powerful approach to find common biological features across many rare conditions. These biological pathways could then become targets for further investigation. Multiple approaches such as functional assays by mass spectrometry on patient-derived reprogrammed neuronal cell types may be needed to validate our bioinformatics findings.

To our knowledge, this is the first time the results of a GO-pathway analysis has been used in a comparative analysis to identify potential biological underpinnings that distinguish IS and other ELE. This approach offers a way to reduce the genetic heterogeneity in IS in a biologically meaningful manner and demonstrates the value of comparative genetic and bioinformatic analysis. This provides a novel biological understanding of ELE, a group of serious diseases for which there is intense interest in harnessing the advances in clinical genomics and developing precision therapies.

## Supporting information

S1 Table epiSEEK® comprehensive sequence analysis of epilepsy and seizure disorders (471 genes). (DOCX)

S2 Table List of genes with pathogenic variants and their association with spasms. (DOCX)

S3 Table Significant enriched gene sets of pathways, cellular compartments and molecular functions and corresponding descriptions, analyses statistical values, and compiled categories, from the mSigDB gene ontology-pathway (GO-pathway) analysis. (DOCX)

S4 Table Evaluations performed in infants with and without spasms. (DOCX)
