# Supplementary material for: Why West? Comparisons of clinical, genetic and molecular features of infants with and without spasms
Source: PLoS One. 2018 Mar 8;13(3):e0193599. doi: 10.1371/journal.pone.0193599 (PMC5843222; doi:10.1371/journal.pone.0193599)
Supplement: S1 Table — (DOCX) [file pone.0193599.s001.docx]

# S1 Table. Courtagen epiSEEK Comprehensive Sequence Analysis of Epilepsy and Seizure Disorders (471 genes)

| *ABAT* | *BCKDK* | *COX10* | *FKTN* | *GPHN* |
| --- | --- | --- | --- | --- |
| *ABCC2* | *BCS1L* | *COX15* | *FLNA* | *GPR55* |
| *ABCC8* | *BRAF* | *CPT1A* | *FLVCR2* | *GPR56* |
| *ACOX1* | *BRAT1* | *CPT2* | *FOLR1* | *GPR98* |
| *ACY1* | *BRD2* | *CSTB* | *FOXG1* | *GRIA3* |
| *ADCK3* | *BTD* | *CTSA* | *FOXH1* | *GRIN1* |
| *ADSL* | *BUB1B* | *CTSD* | *FUCA1* | *GRIN2A* |
| *AGA* | *C12orf57* | *CTSF* | *GABBR1* | *GRIN2B* |
| *AGTR2* | *C12orf65* | *CUL4B* | *GABBR2* | *GUSB* |
| *AHI1* | *CACNA1A* | *CYP2C19* | *GABRA1* | *HCN1* |
| *AKT3* | *CACNA1H* | *CYP2C9* | *GABRA2* | *HCN2* |
| *ALDH4A1* | *CACNB4* | *CYP3A4* | *GABRA3* | *HCN3* |
| *ALDH5A1* | *CASK* | *CYP3A5* | *GABRA4* | *HCN4* |
| *ALDH7A1* | *CASR* | *DAGLA* | *GABRA5* | *HERC2* |
| *ALG1* | *CBL* | *DBT* | *GABRA6* | *HEXA* |
| *ALG11* | *CC2D2A* | *DCLK2* | *GABRB1* | *HEXB* |
| *ALG12* | *CCDC88C* | *DCX* | *GABRB2* | *HGSNAT* |
| *ALG13* | *CCL2* | *DDC* | *GABRB3* | *HNRNPU* |
| *ALG2* | *CDK5RAP2* | *DDOST* | *GABRD* | *HPD* |
| *ALG3* | *CDKL5* | *DEPDC5* | *GABRE* | *HRAS* |
| *ALG6* | *CDON* | *DHCR7* | *GABRG1* | *HSD17B10* |
| *ALG8* | *CENPJ* | *DLD* | *GABRG2* | *HSD17B4* |
| *ALG9* | *CEP152* | *DLGAP2* | *GABRG3* | *HYAL1* |
| *AMT* | *CEP290* | *DNAJC5* | *GABRP* | *IDH2* |
| *APTX* | *CHD2* | *DOLK* | *GABRQ* | *IDS* |
| *ARFGEF2* | *CHRNA2* | *DPAGT1* | *GABRR1* | *IDUA* |
| *ARG1* | *CHRNA4* | *DPM1* | *GABRR2* | *INPP5E* |
| *ARHGEF9* | *CHRNA7* | *DPM3* | *GABRR3* | *IQSEC2* |
| *ARL13B* | *CHRNB2* | *DPYD* | *GALC* | *JRK* |
| *ARSA* | *CLCN2* | *DYRK1A* | *GALNS* | *KANSL1* |
| *ARSB* | *CLCNKA* | *EFHC1* | *GAMT* | *KAT6B* |
| *ARX* | *CLCNKB* | *EFHC2* | *GATM* | *KCNA1* |
| *ASAH1* | *CLN3* | *EIF2B1* | *GCDH* | *KCNAB1* |
| *ASPA* | *CLN5* | *EIF2B2* | *GCSH* | *KCNJ1* |
| *ASPM* | *CLN6* | *EIF2B3* | *GFAP* | *KCNJ10* |
| *ATIC* | *CLN8* | *EIF2B4* | *GJD2* | *KCNJ11* |
| *ATN1* | *CNR1* | *EIF2B5* | *GLB1* | *KCNMA1* |
| *ATP1A2* | *CNR2* | *ELP4* | *GLDC* | *KCNQ2* |
| *ATP1A3* | *CNTN2* | *EMX2* | *GLI2* | *KCNQ3* |
| *ATP2A2* | *CNTNAP2* | *EOMES* | *GLI3* | *KCNT1* |
| *ATP5A1* | *COG1* | *EPM2A* | *GLRA1* | *KCNV2* |
| *ATP6AP2* | *COG4* | *ETFA* | *GLRB* | *KCTD7* |
| *ATP6V0A2* | *COG5* | *ETFB* | *GLUD1* | *KDM5C* |
| *ATP7A* | *COG6* | *ETFDH* | *GLUL* | *KIAA1279* |
| *ATPAF2* | *COG7* | *FAAH* | *GNE* | *KMT2D* |
| *ATR* | *COG8* | *FGD1* | *GNPTAB* | *KRAS* |
| *ATRX* | *COL18A1* | *FGF8* | *GNPTG* | *L2HGDH* |
| *B4GALT1* | *COL4A1* | *FGFR3* | *GNS* | *LAMA2* |
| *BCKDHA* | *COQ2* | *FH* | *GOSR2* | *LARGE* |
| *BCKDHB* | *COQ9* | *FKRP* | *GPC3* | *LBR* |

| *LGI1* | *NODAL* | *POMT2* | *SLC16A2* | *TPP1* |
| --- | --- | --- | --- | --- |
| *LIAS* | *NOTCH3* | *PPT1* | *SLC17A5* | *TREX1* |
| *LIG4* | *NPC1* | *PQBP1* | *SLC19A3* | *TRPM6* |
| *LRPPRC* | *NPC2* | *PRICKLE1* | *SLC1A3* | *TSC1* |
| *MAGI2* | *NPHP1* | *PRICKLE2* | *SLC25A15* | *TSC2* |
| *MAGT1* | *NRAS* | *PRODH* | *SLC25A19* | *TSEN2* |
| *MAP2K1* | *NRXN1* | *PRRT2* | *SLC25A22* | *TSEN34* |
| *MAP2K2* | *OFD1* | *PSAP* | *SLC2A1* | *TSEN54* |
| *MAPK10* | *OPA1* | *PSAT1* | *SLC35A1* | *TUBA1A* |
| *MBD5* | *OPHN1* | *PTCH1* | *SLC35A2* | *TUBA8* |
| *MCOLN1* | *PAFAH1B1* | *PTPN11* | *SLC35C1* | *TUBB2B* |
| *MCPH1* | *PAK3* | *QDPR* | *SLC46A1* | *TUSC3* |
| *ME2* | *PANK2* | *RAB39B* | *SLC4A10* | *UBE3A* |
| *MECP2* | *PAX6* | *RAB3GAP1* | *SLC6A4* | *VANGL1* |
| *MED12* | *PC* | *RAF1* | *SLC6A5* | *VDAC1* |
| *MED17* | *PCDH19* | *RAI1* | *SLC6A8* | *VPS13A* |
| *MEF2C* | *PCNT* | *RARS2* | *SLC9A6* | *VPS13B* |
| *MFSD8* | *PDHA1* | *RBFOX1* | *SMC1A* | *VRK1* |
| *MGAT2* | *PDHX* | *RELN* | *SMC3* | *WDR62* |
| *MGLL* | *PDSS1* | *RFT1* | *SMPD1* | *ZEB2* |
| *MGME1* | *PDSS2* | *RNASEH2A* | *SMS* | *ZIC2* |
| *MLC1* | *PEX1* | *RNASEH2B* | *SNAP25* |  |
| *MMACHC* | *PEX10* | *RNASEH2C* | *SNAP29* |  |
| *MOCS1* | *PEX12* | *RPGRIP1L* | *SNIP1* |  |
| *MOCS2* | *PEX13* | *RTTN* | *SOS1* |  |
| *MOGS* | *PEX14* | *SAMHD1* | *SPRED1* |  |
| *MPDU1* | *PEX16* | *SCARB2* | *SPTAN1* |  |
| *MPI* | *PEX19* | *SCN10A* | *SRD5A3* |  |
| *MTHFR* | *PEX2* | *SCN11A* | *SRPX2* |  |
| *MTOR* | *PEX26* | *SCN1A* | *ST3GAL3* |  |
| *MTR* | *PEX3* | *SCN1B* | *ST3GAL5* |  |
| *MTRR* | *PEX5* | *SCN2A* | *STIL* |  |
| *NAGLU* | *PEX6* | *SCN2B* | *STRADA* |  |
| *NDE1* | *PEX7* | *SCN3A* | *STXBP1* |  |
| *NDUFA1* | *PGK1* | *SCN3B* | *SUCLA2* |  |
| *NDUFA2* | *PGM1* | *SCN4A* | *SUMF1* |  |
| *NDUFAF6* | *PHF6* | *SCN4B* | *SUOX* |  |
| *NDUFS1* | *PHGDH* | *SCN5A* | *SURF1* |  |
| *NDUFS3* | *PIGV* | *SCN7A* | *SYN1* |  |
| *NDUFS4* | *PIK3CA* | *SCN8A* | *SYNGAP1* |  |
| *NDUFS7* | *PIK3R2* | *SCN9A* | *SYP* |  |
| *NDUFS8* | *PLA2G6* | *SCO2* | *TACO1* |  |
| *NDUFV1* | *PLCB1* | *SDHA* | *TBC1D24* |  |
| *NEDD4L* | *PLP1* | *SERPINI1* | *TBX1* |  |
| *NEU1* | *PMM2* | *SETBP1* | *TCF4* |  |
| *NF1* | *PNKP* | *SGCE* | *TGIF1* |  |
| *NGLY1* | *PNPO* | *SGSH* | *TMEM165* |  |
| *NHEJ1* | *POLG* | *SHH* | *TMEM216* |  |
| *NHLRC1* | *POMGNT1* | *SHOC2* | *TMEM67* |  |
| *NIPBL* | *POMT1* | *SIX3* | *TMEM70* |  |
