# Supplementary material for: Why West? Comparisons of clinical, genetic and molecular features of infants with and without spasms
Source: PLoS One. 2018 Mar 8;13(3):e0193599. doi: 10.1371/journal.pone.0193599 (PMC5843222; doi:10.1371/journal.pone.0193599)
Supplement: S2 Table — (DOCX) [file pone.0193599.s002.docx]

**S2 Table. List of genes with pathogenic variants and their association with spasms**

| **GENES** | **Onset of spasms** | | | |
| --- | --- | --- | --- | --- |
|  | **No spasms** | **Spasms at onset** | **Spasms evolved after onset** | **Total** |
| ***ALG13*** | \| 0 \| \| --- \| | \| 1 \| \| --- \| | \| 0 \| \| --- \| | \| 1 \| \| --- \| |
| ***ARX*** | \| 1 \| \| --- \| | \| 1 \| \| --- \| | \| 0 \| \| --- \| | \| 2 \| \| --- \| |
| ***ATP1A3*** | \| 1 \| \| --- \| | \| 0 \| \| --- \| | \| 0 \| \| --- \| | \| 1 \| \| --- \| |
| ***BRAT1*** | \| 1 \| \| --- \| | \| 0 \| \| --- \| | \| 0 \| \| --- \| | \| 1 \| \| --- \| |
| ***CASK*** | \| 1 \| \| --- \| | \| 0 \| \| --- \| | \| 0 \| \| --- \| | \| 1 \| \| --- \| |
| ***CDKL5*** | \| 1 \| \| --- \| | \| 1 \| \| --- \| | \| 2 \| \| --- \| | \| 4 \| \| --- \| |
| ***DCX*** | \| 1 \| \| --- \| | \| 0 \| \| --- \| | \| 0 \| \| --- \| | \| 1 \| \| --- \| |
| ***FBXL4*** | \| 0 \| \| --- \| | \| 1 \| \| --- \| | \| 0 \| \| --- \| | \| 1 \| \| --- \| |
| ***FKTN*** | \| 1 \| \| --- \| | \| 0 \| \| --- \| | \| 0 \| \| --- \| | \| 1 \| \| --- \| |
| ***FOXG1*** | \| 0 \| \| --- \| | \| 0 \| \| --- \| | \| 1 \| \| --- \| | \| 1 \| \| --- \| |
| ***FUCA1*** | \| 0 \| \| --- \| | \| 1 \| \| --- \| | \| 0 \| \| --- \| | \| 1 \| \| --- \| |
| ***GABRG2*** | \| 1 \| \| --- \| | \| 0 \| \| --- \| | \| 0 \| \| --- \| | \| 1 \| \| --- \| |
| ***GLB1*** | \| 1 \| \| --- \| | \| 0 \| \| --- \| | \| 0 \| \| --- \| | \| 1 \| \| --- \| |
| ***GLDC*** | \| 2 \| \| --- \| | \| 0 \| \| --- \| | \| 1 \| \| --- \| | \| 3 \| \| --- \| |
| ***HNRNPR*** | \| 0 \| \| --- \| | \| 1 \| \| --- \| | \| 0 \| \| --- \| | \| 1 \| \| --- \| |
| ***HSD17B4*** | \| 1 \| \| --- \| | \| 0 \| \| --- \| | \| 0 \| \| --- \| | \| 1 \| \| --- \| |
| ***KANSL1*** | \| 0 \| \| --- \| | \| 1 \| \| --- \| | \| 0 \| \| --- \| | \| 1 \| \| --- \| |
| ***KCNQ2*** | \| 2 \| \| --- \| | \| 0 \| \| --- \| | \| 0 \| \| --- \| | \| 2 \| \| --- \| |
| ***KRAS*** | \| 1 \| \| --- \| | \| 1 \| \| --- \| | \| 0 \| \| --- \| | \| 2 \| \| --- \| |
| ***LIS1*** | \| 0 \| \| --- \| | \| 0 \| \| --- \| | \| 2 \| \| --- \| | \| 2 \| \| --- \| |
| ***NDE1*** | \| 1 \| \| --- \| | \| 0 \| \| --- \| | \| 0 \| \| --- \| | \| 1 \| \| --- \| |
| ***NDUFAF5*** | \| 0 \| \| --- \| | \| 1 \| \| --- \| | \| 0 \| \| --- \| | \| 1 \| \| --- \| |
| ***NF1*** | \| 0 \| \| --- \| | \| 1 \| \| --- \| | \| 0 \| \| --- \| | \| 1 \| \| --- \| |
| ***PDHA1*** | \| 0 \| \| --- \| | \| 1 \| \| --- \| | \| 0 \| \| --- \| | \| 1 \| \| --- \| |
| ***PHF8*** | \| 1 \| \| --- \| | \| 0 \| \| --- \| | \| 0 \| \| --- \| | \| 1 \| \| --- \| |
| ***PIGN*** | \| 0 \| \| --- \| | \| 0 \| \| --- \| | \| 1 \| \| --- \| | \| 1 \| \| --- \| |
| ***POLG1*** | \| 1 \| \| --- \| | \| 0 \| \| --- \| | \| 0 \| \| --- \| | \| 1 \| \| --- \| |
| ***POMT2*** | \| 1 \| \| --- \| | \| 0 \| \| --- \| | \| 0 \| \| --- \| | \| 1 \| \| --- \| |
| ***PRRT2*** | \| 4 \| \| --- \| | \| 0 \| \| --- \| | \| 0 \| \| --- \| | \| 4 \| \| --- \| |
| ***RELN*** | \| 1 \| \| --- \| | \| 0 \| \| --- \| | \| 0 \| \| --- \| | \| 1 \| \| --- \| |
| ***SAMHD1*** | \| 1 \| \| --- \| | \| 0 \| \| --- \| | \| 0 \| \| --- \| | \| 1 \| \| --- \| |
| ***SCN1A*** | \| 10 \| \| --- \| | \| 0 \| \| --- \| | \| 0 \| \| --- \| | \| 10 \| \| --- \| |
| ***SCO2*** | \| 0 \| \| --- \| | \| 1 \| \| --- \| | \| 0 \| \| --- \| | \| 1 \| \| --- \| |
| ***SETBP1*** | \| 0 \| \| --- \| | \| 0 \| \| --- \| | \| 1 \| \| --- \| | \| 1 \| \| --- \| |
| ***SLC2A1*** | \| 0 \| \| --- \| | \| 1 \| \| --- \| | \| 0 \| \| --- \| | \| 1 \| \| --- \| |
| ***SMIC1A*** | \| 0 \| \| --- \| | \| 0 \| \| --- \| | \| 1 \| \| --- \| | \| 1 \| \| --- \| |
| ***ST3GAL5*** | \| 1 \| \| --- \| | \| 0 \| \| --- \| | \| 0 \| \| --- \| | \| 1 \| \| --- \| |
| ***STXBP1*** | \| 1 \| \| --- \| | \| 2 \| \| --- \| | \| 0 \| \| --- \| | \| 3 \| \| --- \| |
| ***TRAPPC11*** | \| 1 \| \| --- \| | \| 0 \| \| --- \| | \| 0 \| \| --- \| | \| 1 \| \| --- \| |
| **Trisomy 21 *(DYRK1A, RCAN1, PSMG1, DSCR3, DSCR4)*** | \| 2 \| \| --- \| | \| 15 \| \| --- \| | \| 0 \| \| --- \| | \| 17 \| \| --- \| |
| ***TSC1*** | \| 1 \| \| --- \| | \| 0 \| \| --- \| | \| 0 \| \| --- \| | \| 1 \| \| --- \| |
| ***TSC2*** | \| 2 \| \| --- \| | \| 5 \| \| --- \| | \| 0 \| \| --- \| | \| 7 \| \| --- \| |
| ***UBE3A*** | \| 0 \| \| --- \| | \| 2 \| \| --- \| | \| 0 \| \| --- \| | \| 2 \| \| --- \| |
| ***mt-ATP6*** | \| 0 \| \| --- \| | \| 1 \| \| --- \| | \| 0 \| \| --- \| | \| 1 \| \| --- \| |
| ***mt-ND5*** | \| 0 \| \| --- \| | \| 1 \| \| --- \| | \| 0 \| \| --- \| | \| 1 \| \| --- \| |
| ***tRNA Phe (MT-TF)*** | \| 0 \| \| --- \| | \| 1 \| \| --- \| | \| 0 \| \| --- \| | \| 1 \| \| --- \| |
| **Total** | \| 43 \| \| --- \| | \| 40 \| \| --- \| | \| 9 \| \| --- \| | \| 92 \| \| --- \| |
