# Supplementary material for: Why West? Comparisons of clinical, genetic and molecular features of infants with and without spasms
Source: PLoS One. 2018 Mar 8;13(3):e0193599. doi: 10.1371/journal.pone.0193599 (PMC5843222; doi:10.1371/journal.pone.0193599)
Supplement: S3 Table — (DOCX) [file pone.0193599.s003.docx]

S3 Table. Significant enriched gene sets of pathways, cellular compartments and molecular functions and corresponding descriptions, analyses statistical values, and compiled categories, from the mSigDB gene ontology and pathway analysis

| **Biological Process/Pathways** |  | | | | | | |
| --- | --- | --- | --- | --- | --- | --- | --- |
| **Gene Set Name** | **# Genes in Gene Set (K)** | **Description** | **# Genes in Overlap (k)** | **k/K** | **p‐value** | **FDR q‐value** | **Compiled Pathways used in comparative analyses of children with and without spasms** |
| **BLALOCK_ALZHEIMERS_DISEASE_DN** | 1237 | Genes down‐regulated in brain from patients with Alzheimer's  disease. | 9 | 0.0073 | 7.93E‐06 | 2.37E‐03 | **Alzheimer's disease Signature** |
| **GO_SINGLE_ORGANISM_BEHAVIOR** | 384 | The specific behavior of a single organism in response to external  or internal stimuli. | 8 | 0.0208 | 1.03E‐08 | 1.71E‐05 | **Behavioral Response/Cell Motility to External Stimuli**  **(Locomotion)** |
| **GO_BEHAVIOR** | 516 | The internally coordinated responses (actions or inactions) of  whole living organisms (individuals or groups) to internal or external stimuli. | 8 | 0.0155 | 9.98E‐08 | 1.15E‐04 | **Behavioral Response/Cell Motility to External Stimuli (Locomotion)** |
| **GO_ADULT_BEHAVIOR** | 135 | Behavior in a fully developed and mature organism. | 5 | 0.037 | 4.28E‐07 | 3.04E‐04 | **Behavioral Response/Cell Motility to External Stimuli**  **(Locomotion)** |
| **GO_RESPONSE_TO_ABIOTIC_STIMULUS** | 1024 | Any process that results in a change in state or activity of a cell or an organism (in terms of movement, secretion, enzyme production, gene expression, etc.) as a result of an abiotic (non‐  living) stimulus. | 9 | 0.0088 | 1.72E‐06 | 8.27E‐04 | **Behavioral Response/Cell Motility to External Stimuli (Locomotion)** |
| **GO_ADULT_LOCOMOTORY_BEHAVIOR** | 80 | Locomotory behavior in a fully developed and mature organism. | 4 | 0.05 | 2.00E‐06 | 9.33E‐04 | **Behavioral Response/Cell Motility to External Stimuli**  **(Locomotion)** |
| **GO_LOCOMOTION** | 1114 | Self‐propelled movement of a cell or organism from one location to  another. | 9 | 0.0081 | 3.41E‐06 | 1.34E‐03 | **Behavioral Response/Cell Motility to External Stimuli**  **(Locomotion)** |
| **GO_CELL_MOTILITY** | 835 | Any process involved in the controlled self‐propelled movement of  a cell that results in translocation of the cell from one place to another. | 8 | 0.0096 | 3.67E‐06 | 1.40E‐03 | **Behavioral Response/Cell Motility to External Stimuli (Locomotion)** |
| **GO_RESPONSE_TO_RADIATION** | 413 | Any process that results in a change in state or activity of a cell or an organism (in terms of movement, secretion, enzyme production, gene expression, etc.) as a result of an electromagnetic radiation stimulus. Electromagnetic radiation is a propagating wave in space with electric and magnetic components. These components oscillate at right angles to each other and to the  direction of propagation. | 6 | 0.0145 | 6.50E‐06 | 2.11E‐03 | **Behavioral Response/Cell Motility to External Stimuli (Locomotion)** |
| **GO_CARBOHYDRATE_METABOLIC_PROCESS** | 662 | The chemical reactions and pathways involving carbohydrates, any of a group of organic compounds based of the general formula Cx(H2O)y. Includes the formation of carbohydrate derivatives by the addition of a carbohydrate residue to another molecule. | 9 | 0.0136 | 4.49E‐08 | 6.70E‐05 | **Carbohydrate Metabolism** |
| **GO_CARBOHYDRATE_DERIVATIVE_METABOLIC_PROCESS** | 1047 | The chemical reactions and pathways involving carbohydrate  derivative. | 10 | 0.0096 | 1.98E‐07 | 1.64E‐04 | **Carbohydrate Metabolism** |
| **GO_MONOSACCHARIDE_METABOLIC_PROCESS** | 202 | The chemical reactions and pathways involving monosaccharides, the simplest carbohydrates. They are polyhydric alcohols containing either an aldehyde or a keto group and between three to ten or more carbon atoms. They form the constitutional repeating units of oligo‐ and polysaccharides. | 5 | 0.0248 | 3.11E‐06 | 1.29E‐03 | **Carbohydrate Metabolism** |
| **GO_MANNOSYLATION** | 34 | The covalent attachment of a mannose residue to a substrate  molecule. | 3 | 0.0882 | 7.52E‐06 | 2.30E‐03 | **Carbohydrate Metabolism** |
| **GRAESSMANN_APOPTOSIS_BY_DOXORUBICIN_DN** | 1781 | Genes down‐regulated in ME‐A cells (breast cancer) undergoing  apoptosis in response to doxorubicin [PubChem=31703]. | 14 | 0.0079 | 5.51E‐09 | 1.21E‐05 | **Cell cycle / Cancer‐related / Tumorogenic** |
| **TTGGGAG_MIR150** | 90 | Genes having at least one occurrence of the motif TTGGGAG in their 3' untranslated region. The motif represents putative target (i.e., seed match) of human mature miRNA hsa‐miR‐150 (v7.1  miRBase). | 5 | 0.0556 | 5.63E‐08 | 7.64E‐05 | **Cell cycle / Cancer‐related / Tumorogenic** |
| **MODULE_100** | 544 | Genes in the cancer module 100. | 8 | 0.0147 | 1.49E‐07 | 1.46E‐04 | **Cell cycle / Cancer‐related / Tumorogenic** |
| **MODULE_137** | 546 | CNS genes. | 8 | 0.0147 | 1.53E‐07 | 1.46E‐04 | **Cell cycle / Cancer‐related / Tumorogenic** |
| **MODULE_66** | 552 | Genes in the cancer module 66. | 8 | 0.0145 | 1.67E‐07 | 1.46E‐04 | **Cell cycle / Cancer‐related / Tumorogenic** |
| **DIAZ_CHRONIC_MEYLOGENOUS_LEUKEMIA_UP** | 1382 | Genes up‐regulated in CD34+ [GeneID=947] cells isolated from  bone marrow of CML (chronic myelogenous leukemia) patients, compared to those from normal donors. | 11 | 0.008 | 2.76E‐07 | 2.17E‐04 | **Cell cycle / Cancer‐related / Tumorogenic** |
| **GTAAGAT_MIR200A** | 54 | Genes having at least one occurrence of the motif GTAAGAT in their 3' untranslated region. The motif represents putative target (i.e., seed match) of human mature miRNA hsa‐miR‐200a* (v7.1  miRBase). | 4 | 0.0741 | 4.08E‐07 | 3.04E‐04 | **Cell cycle / Cancer‐related / Tumorogenic** |
| **ENK_UV_RESPONSE_KERATINOCYTE_DN** | 485 | Genes down‐regulated in NHEK cells (normal epidermal  keratinocytes) after UVB irradiation. | 7 | 0.0144 | 1.08E‐06 | 6.47E‐04 | **Cell cycle / Cancer‐related / Tumorogenic** |
| **YAGI_AML_WITH_INV_16_TRANSLOCATION** | 422 | Genes specifically expressed in samples from patients with  pediatric acute myeloid leukemia (AML) bearing inv(16) translocation. | 6 | 0.0142 | 7.35E‐06 | 2.30E‐03 | **Cell cycle / Cancer‐related / Tumorogenic** |
| **GO_ION_TRANSPORT** | 1262 | The directed movement of charged atoms or small charged  molecules into, out of or within a cell, or between cells, by means of some agent such as a transporter or pore. | 10 | 0.0079 | 1.08E‐06 | 6.47E‐04 | **Cellular Ion Transport** |
| **GO_CEREBRAL_CORTEX_DEVELOPMENT** | 105 | The progression of the cerebral cortex over time from its initial formation until its mature state. The cerebral cortex is the outer  layered region of the telencephalon. | 7 | 0.0667 | 2.83E‐11 | 1.41E‐07 | **Cerebral Cortex Development** |
| **GO_CEREBRAL_CORTEX_CELL_MIGRATION** | 43 | The orderly movement of cells from one site to another in the  cerebral cortex. | 4 | 0.093 | 1.61E‐07 | 1.46E‐04 | **Cerebral Cortex Development** |
| **GO_LAYER_FORMATION_IN_CEREBRAL_CORTEX** | 14 | The detachment of cells from radial glial fibers at the appropriate time when they cease to migrate and form distinct layer in the  cerebral cortex. | 3 | 0.2143 | 4.65E‐07 | 3.15E‐04 | **Cerebral Cortex Development** |
| **GO_CEREBRAL_CORTEX_RADIALLY_ORIENTED_CELL_MIGRATION** | 29 | The migration of cells in the developing cerebral cortex in which  cells move from the ventricular and/or subventricular zone toward the surface of the brain. | 3 | 0.1034 | 4.61E‐06 | 1.64E‐03 | **Cerebral Cortex Development** |
| **GO_CENTRAL_NERVOUS_SYSTEM_DEVELOPMENT** | 872 | The process whose specific outcome is the progression of the central nervous system over time, from its formation to the mature structure. The central nervous system is the core nervous system that serves an integrating and coordinating function. In vertebrates it consists of the brain and spinal cord. In those invertebrates with a central nervous system it typically consists of a brain, cerebral  ganglia and a nerve cord. | 13 | 0.0149 | 9.27E‐12 | 1.13E‐07 | **CNS, Head, Neural Stem Cell Development** |
| **GO_HEAD_DEVELOPMENT** | 709 | The biological process whose specific outcome is the progression of a head from an initial condition to its mature state. The head is the  anterior‐most division of the body. | 12 | 0.0169 | 1.52E‐11 | 1.13E‐07 | **CNS, Head, Neural Stem Cell Development** |
| **GO_NEURAL_PRECURSOR_CELL_PROLIFERATION** | 70 | The multiplication or reproduction of neural precursor cells, resulting in the expansion of a cell population. A neural precursor cell is either a nervous system stem cell or a nervous system  progenitor cell. | 4 | 0.0571 | 1.17E‐06 | 6.71E‐04 | **CNS, Head, Neural Stem Cell Development** |
| **GO_LIMBIC_SYSTEM_DEVELOPMENT** | 100 | The progression of the limbic system over time from its initial formation until its mature state. The limbic system is a collection of structures in the brain involved in emotion, motivation and  emotional aspects of memory. | 5 | 0.05 | 9.57E‐08 | 1.15E‐04 | **Development of Pathways/Brain Structures for Learning, Memory, Emotions** |
| **GO_ASSOCIATIVE_LEARNING** | 73 | Learning by associating a stimulus (the cause) with a particular  outcome (the effect). | 4 | 0.0548 | 1.38E‐06 | 7.38E‐04 | **Development of Pathways/Brain Structures for**  **Learning, Memory, Emotions** |
| **GO_FOREBRAIN_DEVELOPMENT** | 357 | The process whose specific outcome is the progression of the forebrain over time, from its formation to the mature structure. The forebrain is the anterior of the three primary divisions of the developing chordate brain or the corresponding part of the adult brain (in vertebrates, includes especially the cerebral hemispheres, the thalamus, and the hypothalamus and especially in higher vertebrates is the main control center for sensory and associative information processing, visceral functions, and voluntary motor  functions). | 8 | 0.0224 | 5.84E‐09 | 1.21E‐05 | **Forebrain Development** |
| **GO_FOREBRAIN_CELL_MIGRATION** | 62 | The orderly movement of a cell from one site to another at least  one of which is located in the forebrain. | 4 | 0.0645 | 7.15E‐07 | 4.64E‐04 | **Forebrain Development** |
| **GO_HIPPOCAMPUS_DEVELOPMENT** | 73 | The progression of the hippocampus over time from its initial  formation until its mature state. | 4 | 0.0548 | 1.38E‐06 | 7.38E‐04 | **Hippocampus Development** |
| **GSE13485_PRE_VS_POST_YF17D_VACCINATION_PBMC_DN** | 200 | Genes down‐regulated in comparison of peripheral blood  mononuclear cells (PBMC) before vs after YF17D vaccination. | 5 | 0.025 | 2.96E‐06 | 1.26E‐03 | **Immunological Signatures: Development & Response**  **Processes** |
| **GSE360_CTRL_VS_T_GONDII_MAC_DN** | 200 | Genes down‐regulated in comparison of macrophages versus  macrophages exposed to T. gondii. | 5 | 0.025 | 2.96E‐06 | 1.26E‐03 | **Immunological Signatures: Development & Response**  **Processes** |
| **GSE43955_10H_VS_60H_ACT_CD4_TCELL_DN** | 200 | Genes down‐regulated in CD4 [GeneID=920] T helper cells Th0: 10h  versus 60h. | 5 | 0.025 | 2.96E‐06 | 1.26E‐03 | **Immunological Signatures: Development & Response**  **Processes** |
| **GO_PROTEIN_COMPLEX_SUBUNIT_ORGANIZATION** | 1527 | Any process in which macromolecules aggregate, disaggregate, or  are modified, resulting in the formation, disassembly, or alteration of a protein complex. | 10 | 0.0065 | 5.88E‐06 | 1.99E‐03 | **Multi‐subunit Protein Complex Formation** |
| **GO_NEURON_MIGRATION** | 110 | The characteristic movement of an immature neuron from germinal zones to specific positions where they will reside as they  mature. | 6 | 0.0545 | 2.70E‐09 | 8.07E‐06 | **Neuronal Migration & Development** |
| **GO_GLIOGENESIS** | 175 | The process that results in the generation of glial cells. This includes the production of glial progenitors and their  differentiation into mature glia. | 5 | 0.0286 | 1.54E‐06 | 7.67E‐04 | **Neuronal Migration & Development** |
| **GO_CELL_MORPHOGENESIS_INVOLVED_IN_NEURON_DIFFERENTIATIO N** | 368 | The process in which the structures of a neuron are generated and organized. This process occurs while the initially relatively unspecialized cell is acquiring the specialized features of a neuron. | 6 | 0.0163 | 3.36E‐06 | 1.34E‐03 | **Neuronal Migration & Development** |
| **PID_LIS1_PATHWAY** | 28 | Lissencephaly gene (LIS1) in neuronal migration and development | 3 | 0.1071 | 4.14E‐06 | 1.54E‐03 | **Neuronal Migration & Development** |
| **GO_NEURON_PROJECTION_MORPHOGENESIS** | 402 | The process in which the anatomical structures of a neuron projection are generated and organized. A neuron projection is any process extending from a neural cell, such as axons or dendrites. | 6 | 0.0149 | 5.57E‐06 | 1.94E‐03 | **Neuronal Migration & Development** |
| **GO_POSITIVE_REGULATION_OF_DENDRITE_MORPHOGENESIS** | 32 | Any process that activates or increases the frequency, rate or  extent of dendrite morphogenesis. | 3 | 0.0938 | 6.24E‐06 | 2.07E‐03 | **Neuronal Migration & Development** |
| **GO_REGULATION_OF_SYNAPTIC_TRANSMISSION_GABAERGIC** | 29 | Any process that modulates the frequency, rate or extent of GABAergic synaptic transmission, the process of communication from a neuron to another neuron across a synapse using the neurotransmitter gamma‐aminobutyric acid (GABA). | 3 | 0.1034 | 4.61E‐06 | 1.64E‐03 | **Neuronal Signaling Processes and Regulation** |
| **GO_SYNAPTIC_SIGNALING** | 424 | Cell‐cell signaling to or from a synapse. | 6 | 0.0142 | 7.55E‐06 | 2.30E‐03 | **Neuronal Signaling Processes and Regulation** |
| **GO_PALLIUM_DEVELOPMENT** | 153 | The process whose specific outcome is the progression of the  pallium over time, from its formation to the mature structure. The pallium is the roof region of the telencephalon. | 7 | 0.0458 | 4.04E‐10 | 1.51E‐06 | **Pallium Development** |
| **GO_TELENCEPHALON_DEVELOPMENT** | 228 | The process whose specific outcome is the progression of the telencephalon over time, from its formation to the mature structure. The telencephalon is the paired anteriolateral division of the prosencephalon plus the lamina terminalis from which the olfactory lobes, cerebral cortex, and subcortical nuclei are derived. | 7 | 0.0307 | 6.49E‐09 | 1.21E‐05 | **Telencephalon Development** |
| **GO_TELENCEPHALON_GLIAL_CELL_MIGRATION** | 20 | The orderly movement of glial cells through the telencephalon. | 3 | 0.15 | 1.45E‐06 | 7.46E‐04 | **Telencephalon Development** |
|  | | | | | | | |

| **Cellular Compartments** |  | | | | | | |
| --- | --- | --- | --- | --- | --- | --- | --- |
| **Gene Set Name** | **# Genes in Gene Set**  **(K)** | **Description** | **# Genes in Overlap (k)** | **k/K** | **p‐value** | **FDR q‐value** | **Compiled Cellular Compartments** |
| **GO_AXON** | 418 | The long process of a neuron that conducts nerve impulses, usually away from the cell body to the terminals and varicosities, which are sites of storage and release of neurotransmitter. | 9 | 0.0215 | 8.49E‐10 | 1.64E‐07 | **Axon** |
| **GO_CELL_PROJECTION** | 1786 | A prolongation or process extending from a cell, e.g. a flagellum or  axon. | 14 | 0.0078 | 5.71E‐09 | 6.92E‐07 | **Axon** |
| **GO_CELL_PROJECTION_PART** | 946 | Any constituent part of a cell projection, a prolongation or process  extending from a cell, e.g. a flagellum or axon. | 11 | 0.0116 | 5.97E‐09 | 6.92E‐07 | **Axon** |
| **GO_AXON_PART** | 219 | A part of an axon, a cell projection of a neuron. | 6 | 0.0274 | 1.65E‐07 | 1.59E‐05 | **Axon** |
| **GO_AXON_INITIAL_SEGMENT** | 12 | Portion of the axon proximal to the neuronal cell body, at the level of the axon hillock. The action potentials that propagate along the  axon are generated at the level of this initial segment. | 2 | 0.1667 | 7.91E‐05 | 3.06E‐03 | **Axon** |
| **GO_CELL_PROJECTION_CYTOPLASM** | 52 | All of the contents of a cell projection, excluding the plasma  membrane surrounding the projection. | 2 | 0.0385 | 1.55E‐03 | 2.80E‐02 | **Axon** |
| **GO_MAIN_AXON** | 58 | The main axonal trunk, as opposed to the collaterals; i.e., excluding  collaterals, terminal, spines, or dendrites. | 2 | 0.0345 | 1.92E‐03 | 3.18E‐02 | **Axon** |
| **GO_KINETOCHORE** | 120 | A multisubunit complex that is located at the centromeric region of  DNA and provides an attachment point for the spindle microtubules. | 4 | 0.0333 | 1.00E‐05 | 6.21E‐04 | **Chromosomal, Cell Division Compartments** |
| **GO_CHROMOSOME_CENTROMERIC_REGION** | 174 | The region of a chromosome that includes the centromeric DNA and associated proteins. In monocentric chromosomes, this region corresponds to a single area of the chromosome, whereas in holocentric chromosomes, it is evenly distributed along the  chromosome. | 4 | 0.023 | 4.32E‐05 | 2.09E‐03 | **Chromosomal, Cell Division Compartments** |
| **GO_CONDENSED_CHROMOSOME_CENTROMERIC_REGION** | 102 | The region of a condensed chromosome that includes the centromere and associated proteins, including the kinetochore. In monocentric chromosomes, this region corresponds to a single area of the chromosome, whereas in holocentric chromosomes, it  is evenly distributed along the chromosome. | 3 | 0.0294 | 2.05E‐04 | 6.25E‐03 | **Chromosomal, Cell Division Compartments** |
| **GO_CHROMOSOMAL_REGION** | 330 | Any subdivision of a chromosome along its length. | 4 | 0.0121 | 5.00E‐04 | 1.16E‐02 | **Chromosomal, Cell Division Compartments** |
| **GO_CONDENSED_CHROMOSOME** | 195 | A highly compacted molecule of DNA and associated proteins  resulting in a cytologically distinct structure. | 3 | 0.0154 | 1.35E‐03 | 2.52E‐02 | **Chromosomal, Cell Division Compartments** |
| **GO_KINESIN_COMPLEX** | 55 | Any complex that includes a dimer of molecules from the kinesin superfamily, a group of related proteins that contain an extended region of predicted alpha‐helical coiled coil in the main chain that likely produces dimerization. The native complexes of several kinesin family members have also been shown to contain additional peptides, often designated light chains as all of the noncatalytic subunits that are currently known are smaller than the chain that contains the motor unit. Kinesin complexes generally possess a force‐generating enzymatic activity, or motor, which converts the free energy of the gamma phosphate bond of ATP  into mechanical work. | 2 | 0.0364 | 1.73E‐03 | 2.95E‐02 | **Chromosomal, Cell Division Compartments** |
| **GO_MICROTUBULE_ASSOCIATED_COMPLEX** | 145 | Any multimeric complex connected to a microtubule. | 3 | 0.0207 | 5.73E‐04 | 1.28E‐02 | **Cytoskeleton, Cell Cortex, Trafficking** |
| **GO_CELL_CORTEX** | 238 | The region of a cell that lies just beneath the plasma membrane and often, but not always, contains a network of actin filaments  and associated proteins. | 3 | 0.0126 | 2.38E‐03 | 3.63E‐02 | **Cytoskeleton, Cell Cortex, Trafficking** |
| **GO_SOMATODENDRITIC_COMPARTMENT** | 650 | The region of a neuron that includes the cell body (cell soma) and  the dendrite, but excludes the axon. | 8 | 0.0123 | 5.72E‐07 | 4.74E‐05 | **Dendrite** |
| **GO_DENDRITE** | 451 | A neuron projection that has a short, tapering, often branched, morphology, receives and integrates signals from other neurons or from sensory stimuli, and conducts a nerve impulse towards the axon or the cell body. In most neurons, the impulse is conveyed from dendrites to axon via the cell body, but in some types of unipolar neuron, the impulse does not travel via the cell body. | 6 | 0.0133 | 1.07E‐05 | 6.21E‐04 | **Dendrite** |
| **GO_ENDOPLASMIC_RETICULUM** | 1631 | The irregular network of unit membranes, visible only by electron microscopy, that occurs in the cytoplasm of many eukaryotic cells. The membranes form a complex meshwork of tubular channels, which are often expanded into slitlike cavities called cisternae. The ER takes two forms, rough (or granular), with ribosomes adhering to the outer surface, and smooth (with no ribosomes attached). | 8 | 0.0049 | 4.05E‐04 | 1.03E‐02 | **Endoplasmic Reticulum** |
| **GO_GOLGI_APPARATUS** | 1445 | A compound membranous cytoplasmic organelle of eukaryotic cells, consisting of flattened, ribosome‐free vesicles arranged in a more or less regular stack. The Golgi apparatus differs from the endoplasmic reticulum in often having slightly thicker membranes, appearing in sections as a characteristic shallow semicircle so that the convex side (cis or entry face) abuts the endoplasmic reticulum, secretory vesicles emerging from the concave side (trans or exit face). In vertebrate cells there is usually one such organelle, while in invertebrates and plants, where they are known usually as dictyosomes, there may be several scattered in the cytoplasm. The Golgi apparatus processes proteins produced on the ribosomes of the rough endoplasmic reticulum; such processing includes modification of the core oligosaccharides of glycoproteins, and the sorting and packaging of proteins for transport to a variety of cellular locations. Three different regions of the Golgi are now recognized both in terms of structure and function: cis, in the vicinity of the cis face, trans, in the vicinity of the trans face, and medial, lying between the cis and trans regions. | 8 | 0.0055 | 1.79E‐04 | 5.78E‐03 | **Golgi** |
| **GO_MITOCHONDRION** | 1633 | A semiautonomous, self replicating organelle that occurs in varying numbers, shapes, and sizes in the cytoplasm of virtually all  eukaryotic cells. It is notably the site of tissue respiration. | 9 | 0.0055 | 6.99E‐05 | 2.89E‐03 | **Mitochondria** |
| **GO_MITOCHONDRIAL_PART** | 953 | Any constituent part of a mitochondrion, a semiautonomous, self replicating organelle that occurs in varying numbers, shapes, and sizes in the cytoplasm of virtually all eukaryotic cells. It is notably  the site of tissue respiration. | 6 | 0.0063 | 6.37E‐04 | 1.32E‐02 | **Mitochondria** |
| **GO_MITOCHONDRIAL_MATRIX** | 412 | The gel‐like material, with considerable fine structure, that lies in the matrix space, or lumen, of a mitochondrion. It contains the enzymes of the tricarboxylic acid cycle and, in some organisms, the  enzymes concerned with fatty acid oxidation. | 4 | 0.0097 | 1.14E‐03 | 2.21E‐02 | **Mitochondria** |
| **GO_MYELIN_SHEATH** | 168 | An electrically insulating fatty layer that surrounds the axons of many neurons. It is an outgrowth of glial cells: Schwann cells supply the myelin for peripheral neurons while oligodendrocytes supply it to those of the central nervous system. | 3 | 0.0179 | 8.78E‐04 | 1.76E‐02 | **Myelin Sheath** |
| **GO_NEURON_PROJECTION** | 942 | A prolongation or process extending from a nerve cell, e.g. an axon  or dendrite. | 13 | 0.0138 | 2.41E‐11 | 1.40E‐08 | **Neural Growth Cone/Projection** |
| **GO_NEURON_PART** | 1265 | Any constituent part of a neuron, the basic cellular unit of nervous tissue. A typical neuron consists of a cell body (often called the soma), an axon, and dendrites. Their purpose is to receive,  conduct, and transmit impulses in the nervous system. | 14 | 0.0111 | 6.68E‐11 | 1.94E‐08 | **Neural Growth Cone/Projection** |
| **GO_SITE_OF_POLARIZED_GROWTH** | 149 | Any part of a cell where non‐isotropic growth takes place. | 4 | 0.0268 | 2.36E‐05 | 1.24E‐03 | **Neural Growth Cone/Projection** |
| **GO_NEURON_PROJECTION_TERMINUS** | 129 | The specialized, terminal region of a neuron projection such as an  axon or a dendrite. | 3 | 0.0233 | 4.08E‐04 | 1.03E‐02 | **Neural Growth Cone/Projection** |
| **GO_CELL_LEADING_EDGE** | 350 | The area of a motile cell closest to the direction of movement. | 4 | 0.0114 | 6.23E‐04 | 1.32E‐02 | **Neural Growth Cone/Projection** |
| **GO_NODE_OF_RANVIER** | 15 | An axon part that is a gap in the myelin where voltage‐gated  sodium channels cluster and saltatory conduction is executed. | 2 | 0.1333 | 1.26E‐04 | 4.55E‐03 | **Node of Ranvier** |
| **GO_CATALYTIC_COMPLEX** | 1038 | A protein complex which is capable of catalytic activity. | 7 | 0.0067 | 1.43E‐04 | 4.88E‐03 | **Plasma Membrane** |
| **GO_TRANSPORTER_COMPLEX** | 321 | A protein complex facilitating transport of molecules (proteins, small molecules, nucleic acids) into, out of or within a cell, or  between cells. | 4 | 0.0125 | 4.51E‐04 | 1.09E‐02 | **Plasma Membrane** |
| **GO_INTRINSIC_COMPONENT_OF_PLASMA_MEMBRANE** | 1649 | The component of the plasma membrane consisting of the gene products and protein complexes having either part of their peptide sequence embedded in the hydrophobic region of the membrane or some other covalently attached group such as a GPI anchor that  is similarly embedded in the membrane. | 7 | 0.0042 | 2.20E‐03 | 3.54E‐02 | **Plasma Membrane** |
| **GO_CYTOPLASMIC_REGION** | 287 | Any (proper) part of the cytoplasm of a single cell of sufficient size  to still be considered cytoplasm\ | 4 | 0.0139 | 2.95E‐04 | 8.16E‐03 | **Soma/Cell Body** |
| **GO_SYNAPSE** | 754 | The junction between a nerve fiber of one neuron and another neuron or muscle fiber or glial cell; the site of interneuronal communication. As the nerve fiber approaches the synapse it enlarges into a specialized structure, the presynaptic nerve ending, which contains mitochondria and synaptic vesicles. At the tip of the nerve ending is the presynaptic membrane; facing it, and separated from it by a minute cleft (the synaptic cleft) is a specialized area of membrane on the receiving cell, known as the postsynaptic membrane. In response to the arrival of nerve impulses, the presynaptic nerve ending secretes molecules of neurotransmitters into the synaptic cleft. These diffuse across the  cleft and transmit the signal to the postsynaptic membrane. | 8 | 0.0106 | 1.73E‐06 | 1.25E‐04 | **Synapse** |
| **GO_SYNAPSE_PART** | 610 | Any constituent part of a synapse, the junction between a nerve fiber of one neuron and another neuron or muscle fiber or glial  cell. | 6 | 0.0098 | 5.79E‐05 | 2.58E‐03 | **Synapse** |
| **GO_PRESYNAPSE** | 283 | The part of a synapse that is part of the presynaptic cell. | 4 | 0.0141 | 2.80E‐04 | 8.12E‐03 | **Synapse** |
| **GO_CELL_JUNCTION** | 1151 | A cellular component that forms a specialized region of connection between two or more cells or between a cell and the extracellular matrix. At a cell junction, anchoring proteins extend through the plasma membrane to link cytoskeletal proteins in one cell to cytoskeletal proteins in neighboring cells or to proteins in the  extracellular matrix. | 6 | 0.0052 | 1.68E‐03 | 2.95E‐02 | **Synapse** |
| **GO_TERMINAL_BOUTON** | 64 | Terminal inflated portion of the axon, containing the specialized apparatus necessary to release neurotransmitters. The axon terminus is considered to be the whole region of thickening and  the terminal bouton is a specialized region of it. | 2 | 0.0312 | 2.33E‐03 | 3.63E‐02 | **Synapse** |
|  | | | | | | | |

| **Molecular Functions** |  | | | | | | |
| --- | --- | --- | --- | --- | --- | --- | --- |
| **Gene Set Name** | **# Genes in**  **Gene Set (K)** | **Description** | **# Genes in Overlap (k)** | **k/K** | **p‐value** | **FDR q‐value** | **Compiled Molecular Functions** |
| **GO_IDENTICAL_PROTEIN_BINDING** | 1209 | Interacting selectively and non‐covalently with an identical protein  or proteins. | 8 | 0.0066 | 5.23E‐05 | 2.36E‐02 | **Identical Protein Binding** |
| **GO_MACROMOLECULAR_COMPLEX_BINDING** | 1399 | Interacting selectively and non‐covalently with any  macromolecular complex. | 9 | 0.0064 | 2.10E‐05 | 1.90E‐02 | **Macromolecular Interaction** |
| **GO_TRANSFERASE_ACTIVITY_TRANSFERRING_PHOSPHORUS_CONTAINI**  **NG_GROUPS** | 992 | Catalysis of the transfer of a phosphorus‐containing group from  one compound (donor) to another (acceptor). | 7 | 0.0071 | 1.08E‐04 | 3.25E‐02 | **Phosphorylation** |
