# Supplementary material for: Why West? Comparisons of clinical, genetic and molecular features of infants with and without spasms
Source: PLoS One. 2018 Mar 8;13(3):e0193599. doi: 10.1371/journal.pone.0193599 (PMC5843222; doi:10.1371/journal.pone.0193599)
Supplement: S4 Table — (DOCX) [file pone.0193599.s004.docx]

**S4 Table. Evaluations performed in infants with and without spasms.**

|  | **Overall** | |  |
| --- | --- | --- | --- |
|  | **Spasms (N=253)** | **No spasms (N=256)** |  |
| **Any imaging** | 246 (97%) | 238 (93%) | 0.02 |
| **MRI** | 245 (97%) | 234 (91%) | 0.009 |
| **Metabolic testing** |  |  |  |
| **Blood** | 187 (74%) | 139 (54%) | <0.0001 |
| **Urine** | 137 (54%) | 96 (38%) | 0.0002 |
| **CSF** | 71 (28%) | 64 (25%) | 0.43 |
|  |  |  |  |
|  | **Non-acquired injuries** | |  |
|  | **Spasms (N=200)** | **No spasms (n=230)** |  |
| **Genetic testing** |  |  |  |
| **Any testing** | 144 (72%) | 107 (47%) | <0.0001 |
| **Classical Karyotyping** | 31 (16%) | 17 (7%) | 0.008 |
| **Chromosome Micro Array** | 81 (41%) | 64 (28%) | 0.006 |
| **Epilepsy gene panel** | 55 (28%) | 35 (15%) | 0.002 |
| **Whole Exome Sequencing** | 16 (8%) | 12 (5%) | 0.24 |
| **Mitochondrial** | 11 (6%) | 4 (2%) | 0.03 |
| **Other*** | 37 (19%) | 29 (13%) | 0.09 |

* Included tests of individual or a few targeted genes and specialized gene sequencing panels
